# ORMOSIL Coatings Enriched with CeO_2_ (5-ATDT)-Ceramic Nanocontainers for Enhanced Protection of HDG Steel Used in Concrete

**DOI:** 10.3390/ma15113913

**Published:** 2022-05-31

**Authors:** George Kordas

**Affiliations:** Self-healing Structural Materials Laboratory, Peter the Great St. Petersburg Polytechnic University, 195 251 St. Petersburg, Russia; gckordas@gmail.com

**Keywords:** ORMOSIL, nanocontainers, corrosion, inhibitors, steel

## Abstract

This paper reports developing an innovative method of anticorrosion protection based on organically modified silica (ORMOSIL) enriched with CeO_2_ ceramic nanocontainers loaded with 5-amino-1, 3, 4-thiadiazole-2-thiol (5-ATDT) on hot-dip galvanized zinc (HDG) steel used to strengthen cement in concrete. The chemistry of ORMOSIL coatings and the production of CeO_2_ ceramic nanocontainers are described in detail for reproduction by other researchers. The anticorrosion properties of these novel coatings were investigated through frequency response analysis (FRA). As a result, the coatings HDG-ORMOSIL + CeO_2_ (5-ATDT) were better than the samples of HDG steel, HDG-ORMOSIL, and HDG-ORMOSIL + CeO_2_ (EMPTY) by a factor of 1033.60, 109.21, and 7.76 in terms of anticorrosion protection, respectively.

## 1. Introduction

The term corrosion is accredited to various definitions. According to corrosion science, corrosion relates to the reaction of a solid to its environment. According to corrosion engineering, corrosion relates to the interaction of a metal with its environment. The result is the degradation of the function of the metal due to a change in its properties. The term corrosion refers to the process and not the result of corrosion damage, wear and tear. The importance of corrosion is significant and determined by its consequences on the economy of the countries [1]. A frequent example of corrosion is reinforced concrete on bridges and highways and the destruction of steel structures in factories. It is frequent in refrigerator heat exchangers, shipbuilding, food industry, transport, space exploration, and defense systems. The application of corrosion protection methods is imperative for economic reasons, enhancing the safety of structures and equipment and safeguarding the waste of resources. Due to the exceptional mechanical properties of steel and the ease of formatting, and its low cost, it is widely used in construction. However, its receptiveness to corrosion in the presence necessarily requires some form of protection. Applying an appropriate protection method aims to reduce corrosion rate by changing the thermodynamic or kinetic corrosion parameters.

In the recent past, one used chromium (VI) to protect metals. However, due to the carcinogenic consequences of Cr (VI), there has been a great need to develop new protection methods based on polymeric coatings that are friendly to the environment and humans [2]. Just recently, the polymeric coatings were enriched with nanocontainers loaded with inhibitors. This technology works well for aluminum and magnesium metals [3,4,5]. In some cases, these coatings are multifunctional because they exhibit self-healing, antifouling, and antimicrobial protection [4,6,7,8,9]. Recent publications describe different technologies for developing protective coatings based on LDH-type nanocontainers [10]. Combining the two nanocontainer systems, e.g., CeMo and LDH, enhances metals’ protection.

Hot-dip galvanized zinc (HDG) coating is ideal for protection against corrosion of steel bars in concrete structures used in marine environments [11]. The recently established code and standard reflect the importance of using galvanized steel for corrosion protection [12]. A marine environment causes high corrosion rates of unprotected steel amounting to ~500 mm per year [13]. This corrosion environment will destroy the marine structures exposed to these environments after fifteen years of service. The literature, however, has reported good corrosion performance for rods coated with zinc. One uses these metals on decks of bridges and various marine structures under adverse climatic conditions [11,14]. From these works, galvanized steel would provide similar improvement after concrete preparation following coarse aggregates. During the hardening of concrete at a very high pH, hydrogen and zinc corrosion products are created, resulting in the loss of strength of the bonds between concrete and galvanized rod [15]. Bird reports that hydrogen evolution does not occur on pure zinc surfaces where iron and zinc are in contact [16]. The corrosion reaction of a part of the pure zinc layer is responsible for the passivity of galvanized steel [17]. The beneficial effect on the properties of concrete with smooth galvanized rods may be due to the formation of corrosion products resulting from the dissolution of clean zinc layers [11,18]. While zinc tends to dissolve in acidic aqueous solutions, neutral or very alkaline solutions with the evolution of hydrogen, on the contrary, in alkaline solutions (pH values between 8.5 and 10.5), hydroxide film occurs, and corrosion arises only at a meager rate [11].

This work harmonizes the established self-healing technology recently developed in our laboratory on a large type of HDG steel metal using ORMOSIL technology enriched with CeO_2_ ceramic nanocontainers filled with 5-amino-1, 3, 4-thiadiazole-2-thiol (5-ATDT) [3,19,20,21]. Further, the properties of the coatings were evaluated by frequency response analysis (FRA), scanning electron microscopy (SEM), and X-ray diffraction methods. Finally, for the first time, the chemistry of the coatings was described in detail, and there is an emphasis to prove the self-healing phenomenon in our samples based on our nanocontainer technology. 

## 2. Materials and Methods

### 2.1. Chemicals

In this paper, analytical grade chemicals were used such as absolute ethanol (Sigma-Aldrich, St. Louis, MO, USA), *N*-(2-aminoethyl)-3-(trimethyloxypyrite) propylamine (*N*-(2-Aminoethyl)-3-(trimethoxysilyl) propylamine, Ζ 6020, Sigma-Aldrich, St. Louis, MO, USA), epoxy resin (epoxy resin “Araldite GY 257”, GY 257, Ciba-Geigy, Basel, Switzerland), and 2,2′-diaminodyethylamine (2,2′-Diaminodiethylamine, HS 943, Sigma-Aldrich, St. Louis, MO, USA). In addition, the chemicals 5-amino-1, 3, 4-thiadiazole-2-thiol (5-ATDT), and Cerium(III) acetylacetonate were purchased by Acros-Organics, Morris Plains, NJ, USA. 

The ORMOSIL coatings were produced by using absolute ethanol (Sigma-Aldrich, St. Louis, MO, USA), *N*-(2-aminoethyl)-3-(trimethyloxypyrite) propylamine, Ζ 6020, (Sigma-Aldrich, St. Louis, MO, USA), epoxy resin (epoxy resin “Araldite GY 257”, GY 257, Ciba-Geigy, Basel, Switzerland), 2,2′-Diaminodiethylamine, HΥ 943, (Sigma-Aldrich, St. Louis, MO, USA). 

### 2.2. Instrumentation

A scanning electron (SEM) microscope (Thermo Fisher Scientific, FEI, Hillsboro, OR, USA)was used with an FEI Inspect microscope that works on 25 kV. The X-ray Diffraction (XRD) measurements were conducted with a powder crystallographer of the SIEMENS D-500 equipped with a CuKa wave lamp of 1.5418 Å. The thermogravimetric analysis (TGA) measurements were performed with a Perkin Elmer (Pyris Diamand S II) analyzer (Agilent, Santa-Clara, CA, USA) heating at a rate of 10 °C min^−1^ in the presence of ambient air. The electrochemical behavior of the coatings was investigated with a frequency response analysis (FRA) Solartron ModuLab XM MTS system acquired using the ModuLab System operating and analysis software (Solartron, AMETEK, Version 2.1.5302 2014, Wokingham, UK). This equipment was connected to an electrochemical cell made of plexiglass with dimensions of about 10 × 10 × 10 cm^3^. We examined the sample on one vertical wall of the electrochemical cell communicating with the 0.5 M NaCl solution via a circular opening hole (1 cm^2^). The samples were exposed to the above salt solution for 30, 60, 90, and 180 h at ambient temperature and evaluated electrochemically. The electrochemical cell also housed a flat platinum foil with 1 cm × 1 cm and a reference electrode (RE) in saturated solution KCl (SCE Hg/HgCl saturated KCl).

### 2.3. Surface Treatment

Each substrate is cleaned before use to remove fats and greases resulting from the industrial process of producing the alloy and the passive layer of oxides. The present samples were cleaned in acetone for 10 min, were then wiped with filter paper, and afterward immersed in NaOH (pH = 11) alkaline solution for 4 to 5 min at a temperature of 50 °C. In the end, the samples were rinsed with distilled water and then dried in the air.

### 2.4. Nano Container Production

The production of the nanocontainers involves three steps [20,21,22,23,24]. The first is the creation of a spherical polystyrene template. The second stage is coating the template with the oxide to create a shell. The third step concerns the removal of the template by burning it to create empty nanospheres.

#### 2.4.1. Polystyrene Nanospheres

An emulsion polymerization method was used for the manufacture of polystyrene nanospheres. The styrene was distilled twice before use under reduced pressure. Table 1 gives the conditions of production that took place in a reactor of 500 cm^3^. Oxygen in the solution was eliminated via purification with argon gas before the polymerization process began. Polymerization lasted 12 h. The polymer solution was first centrifuged, then discarded the supernatant liquid, and the sediment was rinsed with distilled water.

**Figure 1:**
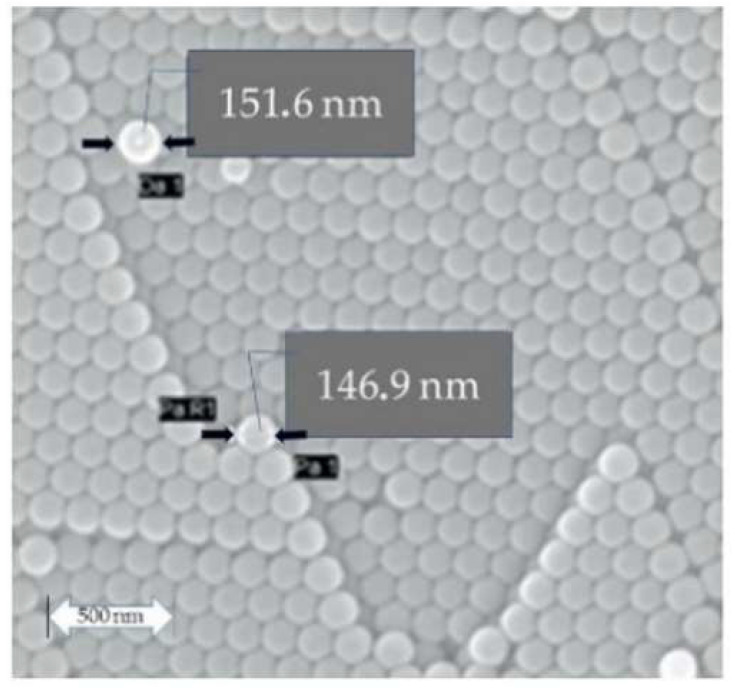
SEM micrograph of polystyrene nanospheres produced under the conditions of Table 1.

Figure 1 shows the SEM micrograph of the polystyrene nanospheres with a uniform shape. The nanospheres were coated with gold to make them conductive and observable with the SEM. The total diameter of polystyrene nanospheres was equal to 150 +/− 10 nm with these procedures. Here, one needs to remove the additional diameter of about 15 nm resulting from the gold coating to obtain the actual diameter of polystyrene nanospheres.

#### 2.4.2. Emulsion Polymerization

The polystyrene nanosphere must be negatively charged to bind cerium, accomplished using emulsion polymerization. For this purpose, a water-soluble potassium persulfate (KPS) reagent was used as the inceptor to polymerize styrene monomers. First, the sodium dodecyl sulfate (SDS) emulsifier is added to the water to create micelles, and then the monomer styrene is added. A small amount of monomer penetrates the micelles and inflates them, while the remaining amount of monomer forms pellets stabilized in the solution by the molecules of the emulsifier. Then, the water-soluble inceptor (KPS) was added to the solution.

At a temperature higher than 80 °C, KPS gives negatively charged sulfate roots (SO_4_^−^) which act as an antiquated polymerization. These roots penetrate the mixed (the micelles are 10^6^–10^8^ times larger than the pellets of the monomers), and polymerization begins.

Styrene monomer was chosen for polymerization because it develops polystyrene roots {^–^O_4_S- [-CH_2_-CH (C_6_H_5_)-] _n_-CH_2_-CH (C_6_H_5_)^–^} which end with a charge. The reactions that take place during polymerization are the following: 

Initiation of Polymerization

K_2_S_2_O_8_  →  2K^+^ + S_2_O_8_^−2^

S_2_O_8_^−2^  →  2 SO_4_^−^

SO_4_**^−^** + CH_2_ = CH(C_6_H_5_)   →  ^–^O_4_S-CH_2_-CH(C_6_H_5_)

Propagation of Polymerization

^–^O_4_S-CH_2_-CH(C_6_H_5_) + n CH_2_ = CH(C_6_H_5_)

^–^O_4_S-[-CH_2_-CH(C_6_H_5_)-]  _n_-CH_2_-CH(C_6_H_5_)

End Polymerization

^–^O_4_S-[-CH_2_-CH(C_6_H_5_)-]  _n_-CH_2_-CH(C_6_H_5_) +

^–^O_4_S-[-CH_2_-CH(C_6_H_5_)-]_m_-CH_2_-CH(C_6_H_5_)

^–^O_4_S-[-CH_2_-CH(C_6_H_5_)-]  _n+m+2_  -SO_4_^–^

#### 2.4.3. Polystyrene Nanosphere Coating

The polystyrene nanospheres were coated through the sol–gel method to form a cerium oxide (CeO_2_) layer using aqueous solution Ce (acac) _3_ and PVP, as shown in Table 2. Next, the solution was aged, centrifuged, and washed using distilled water. 

**Figure 2:**
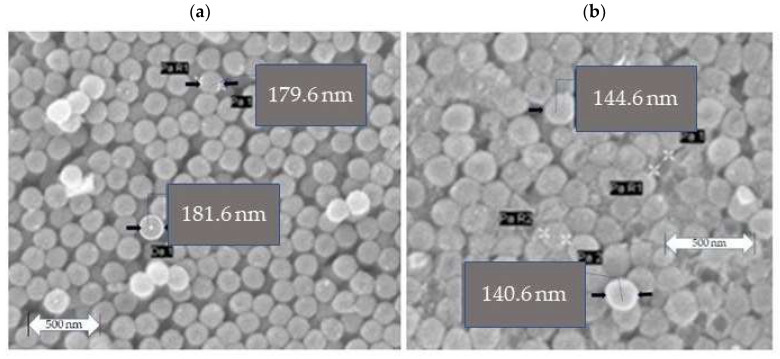
Polystyrene spheres coated with Ce (acac)_3_ resulted in a total sphere size of 180 +/− 10 nm (**a**). SEM of CeO_2_ nanocontainers after the second heat treatment at 600 °C (**b**).

Figure 2a shows the SEM micrograph of the CeO_2_ coated polystyrene. The coating of polystyrene nanospheres with Ce (acac) _3_ gives nanospheres 180 +/− 10 nm, including gold coating.

#### 2.4.4. Production of Empty Nanocontainers

The last stage of the production of nanospheres is the removal of polystyrene nuclei by sintering. The coated nanospheres were placed in clock glass, dried at room temperature, and then at 60 °C. The composite material was then heat-treated for 4 h at 600 °C. The heating speed was 10 °C min^−1^. Table 3 gives the diameters of the CeO_2_ nanospheres after the first (Figure 2a) heat treatment described above and after a second (Figure 2b) repeated heat treatment with the same conditions.

Figure 2b shows the CeO_2_ nanospheres after the second heat treatment.

#### 2.4.5. X-ray Crystallography

**Figure 3:**
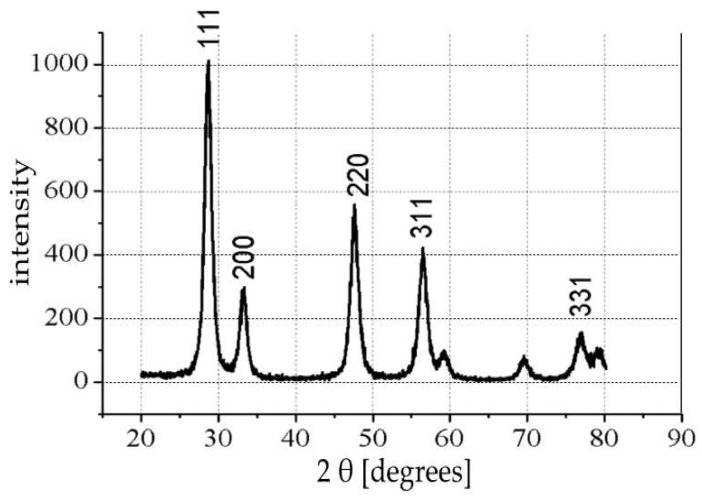
XRD pattern of the CeO_2_ ceramic nanocontainers heat-treated at 600 °C.

Figure 3 shows the XRD pattern of the CeO_2_ nanocontainers indicating the position of the peaks and their intensity. Comparing these peaks with the peaks of crystalline CeO_2_ cerianite (Library: JCPDS-ICDD 1997 International Center for Diffraction Data) suggests the formation of crystalline cerianite sintering the coated nanospheres at the 600 °C. The XRD pattern suggests of formation of pure CeO_2_ nanocontainers. 

#### 2.4.6. Filling Nanocontainers with Corrosion Inhibitor

The nanocontainers prepared were then filled with a corrosion inhibitor such as 5-amino-1, 3, 4-thiadiazole-2-thiol (5-ATDT). First, the corrosion inhibitor was dissolved in an acetone solvent to produce a saturated solution. Next, a small number of nanocontainers was placed in a sealed container. The air is removed from the container and, consequently, from the nanocontainers with a vacuum system. Then, the saturated solution with the corrosion inhibitor enters the sealed container, stirring the whole mixture at room temperature for 12 h. Finally, the nanocontainers were filled with an inhibitor, and the loaded nanocontainers were collected via centrifugation, followed by a vacuum drying process for 12 h.

**Figure 4:**
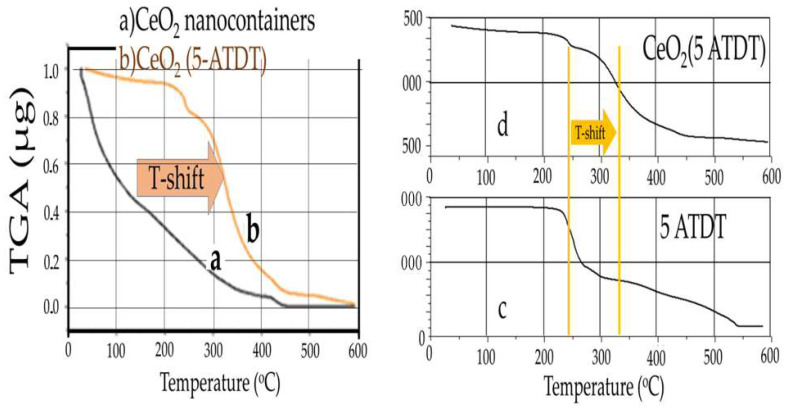
TGA diagrams of (**b**,**d**) CeO_2_ (5-ATDT), (**a**) CeO_2_ (empty) nanocontainers and (**c**) 5-ATDT alone. T-Shift due to delayed burning of 5-ATDT, the weight loss corresponds to the loaded mass into the nanocontainers.

Figure 4 shows the TGA diagram of cerium oxide nanostructures filled with corrosion inhibitors. Additionally, the exact figure shows the corresponding normalized diagram of 5-ATDT compared to empty nanocontainers.

This diagram shows sudden weight losses corresponding to the oxidative degradations of the corrosion inhibitor (Figure 4c). Comparing the normalized TGA charts, one observes that nanocontainers filled with corrosion inhibitors show a weight change at higher temperatures than empty nanocontainers (Figure 4a,b). This delay in combustion is attributed to the inhibitor loaded into the nanocontainers. For example, in CeO_2_ nanocontainers loaded with 5-ATDT, the % loss of mass between 200 °C and 550 °C is 21, 91%, or 2439 μg. 

#### 2.4.7. ORMOSIL Synthesis

Table 4 includes the number of chemicals used for the composition of ORMOSIl coatings [25]. The process for preparing the coating that includes nanocontainers loaded with 5-ATDT includes five steps. First, Z 6020 hydrolyzes in absolute ethanol for 1 h (solution A). Then, the resin GY 257 is dissolved in absolute ethanol (solution B). Then, A and B solutions are mixed to form solution C. Then, HR 943 was dissolved in 25 mL acetone (D solution). Finally, the solutions C and D were mixed and stirred for 8 h. Finally, the filled nanocontainers were added to the above solution, under vigorous stirring, 1 h before the immersion process, and in a concentration of 1% *w*/*w*. 

HDG frames were dipped in the solution containing epoxy resin -ORMOSIL-nanocontainers six times at a 32 cm/min speed. The frames remain in the solution for 1 min. Then, the coated frames undergo aging treatment at a temperature of 70 °C for four days. The following nomenclature was used in the following discussion: the coating without additives (nanocontainers empty or filled) was named HDG-ORMOSIL, and the coating containing empty nanocontainers was named HDG-ORMOSIL + CeO_2_ (EMPTY), and finally, the coating containing filled nanocontainers with corrosion inhibitor was named HDG-ORMOSIL + CeO_2_ (5-ATDT). SEM was used to determine the thickness of the coatings in the ORMOSIL CeO_2_ (EMPTY) and ORMOSIL CeO_2_ (5-ATDT) coatings exposed the two samples to a solution of 0.5 M NaCl at ambient temperature for eight days was about ~500 μm. 

**Figure 5:**
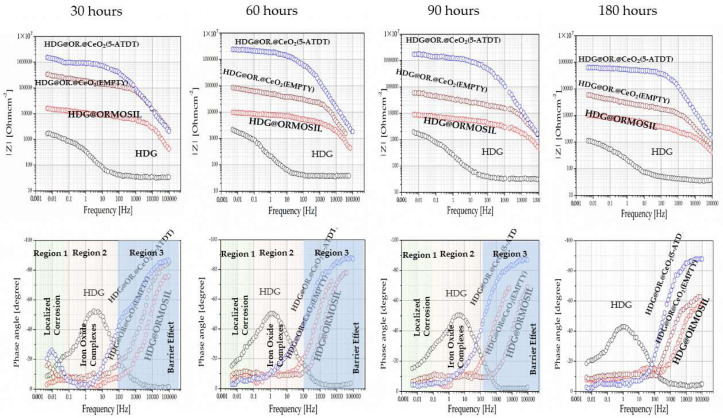
EIS bode sample diagrams: uncoated galvanized steel, HDG-ORMOSIL, HDG-ORMOSIL + CeO_2_ (EMPTY), HDG-ORMOSIL + CeO_2_ (5-ATDT) after exposure to a solution of 0.5 M NaCl at ambient temperature for 30, 60, 90, and 180 h.

The test involves exposing the coated frames to a solution of 0.5 M NaCl for 30, 60, 90, and 180 h. The EIS assessed the coated samples’ protective capabilities and corrosion mechanism. Figure 5 shows the EIS Bode plots of all coatings that offer high anticorrosion protection to the HDG steel alloy. The R_p_ of the bare HDG steel, ORMOSIL HDG steel, ORMOSIL plus CeO_2_ (empty) HDG steel, and ORMOSIL plus CeO_2_ (5-ATDT) HDG steel were 1.62 × 10^3^, 1.54 × 10^4^, 21.62 × 10^4^, and 16.78 × 10^5^ Ohm cm^2^, respectively [26]. One can calculate that the R_p_ of ORMOSIL HDG steel, ORMOSIL CeO_2_ (empty) HDG steel, and ORMOSIL CeO_2_ (5-ATDT) HDG steel were 7.76, 109.21, and 1033.60 times greater than the bare metal, respectively [26]. These outcomes propose that the deposition of the abovementioned coatings on an alloy HDG steel upsurges protection against corrosion of the alloy due to barrier effect properties. The barrier effect increases in the ORMOSIL CeO_2_ (5-ATDT) HDG sample due to added inhibitor release from the CeO_2_ nanocontainers. It is also mentioned that the coating ORMOSIL containing 5-ATDT is a bit better (4.0 × 10^4^ Ohm) than the one that is without 5-ATDT (1.54 × 10^4^ Ohm) but much worse than the one it contains CeO_2_ (5-ATDT) (16.78 × 10^4^ Ohm). One can explain this value to entrapment of 5-ATDT in ORMOSIL and is not free to act to source the metal from corrosion as much as the 5-ATDT built into the CeO_2_ nanocontainers. This result proves the usefulness of nanocontainers for their anticorrosion protection. In the diagrams showing the phase with the measurement frequency, observing different behavior between the samples. The spectrum into three areas, 1, 2, and 3, attributed to localized corrosion, iron oxide complexes, and barrier effect [5].

**Figure 6:**
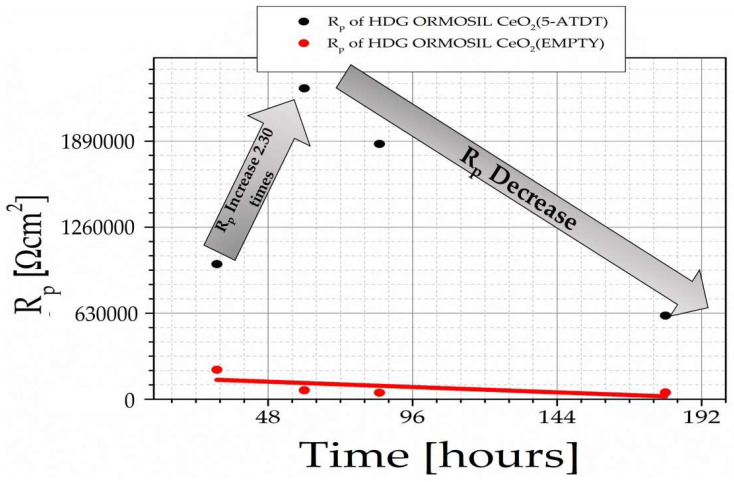
R_p_ of HDG-ORMOSIL + CeO_2_ (EMPTY) and HDG-ORMOSIL + CeO_2_ (5-ATDT) function of exposure to the salt solution.

Figure 6 shows the R_p_ of HDG-ORMOSIL + CeO_2_ (EMPTY) and HDG-ORMOSIL + CeO_2_ (5-ATDT) after exposure to a solution of 0.5 M NaCl at ambient temperature for 30, 60, 90, and 180 h.

## 3. Discussion

HDG steel, among others, is used widely to strengthen concrete in harsh environments [15,18,27,28]. It is of great importance to improve the anticorrosion performance in marine environments. This paper’s scope extensively described the chemistry that evolved for ORMOSIL coatings for other investigators to reproduce our results. During its use in the concrete, ORMOSIL coatings reduce the corrosion phenomena, as demonstrated by our measurements. Furthermore, these ORMOSIL coatings offer significant corrosion protection to metal substrates (Figure 6). Furthermore, our team developed, for the first time in the literature, ceramic nanocontainers for the additional protection of metals filled with corrosion inhibitors, in this case, 5-ATDT. Here, a simple system of ceramic nanocontainers was presented such as CeO_2_.

From the XRD diagram (Figure 3), the CeO_2_ nanocontainers consist of a pure crystalline phase of CeO_2_ cerianite. For the first time, the existence of a vacuum device was disclosed and developed in the laboratory for filling the nanocontainers with corrosion inhibitors. The TGA measurements reveal that the occupancy rate in 5-ATDT in nanocontainers reaches a percentage corresponding to 2439 μg. This quantity is released on demand to impinge protection to metals against corrosion in the sample HDG-ORMOSIL + CeO_2_ (5-ATDT) compared to samples of galvanized steel, HDG-ORMOSIL, and HDG-ORMOSIL + CeO_2_ (EMPTY). The protection increase is 7.76, 109.21, and 1033.60 times more tremendous in HDG-ORMOSIL + CeO_2_ (EMPTY), HDG-ORMOSIL, and bare metal. As far as phase angle measurements are concerned (Figure 5), the results are fascinating and characteristic of the performance of ORMOSIL coatings charged with corrosive inhibitors [5]. The unprotected metal ORMOSIL exhibits corrosive reactions on its surface limited to range 2 of our measurement. The literature reports the formation of zinc corrosion products into solutions extensively. For example, ZnO and plates of β-Zn(OH)_2_ cover the surface of a zinc sample when placed in a solution of 0.5 M NaCl for several days; one observes craters up to 1 mm in size free oxides and hydroxides [17,29]. Chlorine products occur in the middle of the crater that fills the hole. β-Zn(OH)_2_ covers the surface after a prolonged exposure period. The inner wall around the crater also consists of chloride hydroxide, then follows β-Zn(OH)_2,_ partly covered by ZnO, and finally by a zone of β-Zn(OH)_2_ [29].

The ORMOSIL, ORMOSIL + CeO_2_ (EMPTY), and ORMOSIL + CeO_2_ (5-ATDT) form a barrier, which in the last sample is more pronounced. Therefore, in the following, the ORMOSIL + CeO_2_ (EMPTY) and ORMOSIL + CeO_2_ (5-ATDT) coatings are emphasized and measured after exhibiting to the salt solution for 30, 60, 90, and 180 h. First, one notices that the ORMOSIL + CeO_2_ (EMPTY) exhibits a clear improvement in the ORMOSIL coating (Figure 5). There is quite a wealth of bibliography that examines this topic in coatings that exist with scattered cerium, but there are also many conflicting interpretations that are not the subject of this work to solve or discuss here. Nevertheless, the conclusion from these studies is the contribution of cerium that reacts with oxygen as much as hydrogen peroxide can act as oxidizing agents that produce Ce^4+^ and CeO_2_:

Ce^3+^ + 2H_2_O ⇋ Ce (OH)_2_^2+^ + 2H^+^ + e^−^ (1)

Ce (OH) _2_^2+^ ⇋ CeO_2_ + 2H^+^ (2)

In the present conditions, hydrogen peroxide causes the oxidation of Ce^4+^ [30]. Given the above discussion, one can assume the formation of Ce-rich film as observed by many others in the literature [31]. Figure 6 suggests that cerium depletes linearly with the time from the ORMOSIL coatings to the time of exposure to the salt solution. Figure 5 shows a different behavior of the ORMOSIL coatings with CeO_2_ (5-ATDT) concerning the ORMOSIL coatings with empty CeO_2_ nanocontainers. The 5-ATDT entrapped in the CeO_2_ nanocontainers released from them as time exposure in the salt solution increased accounts for the other performance. The anticorrosion performance first increases with the time up to 60 h of exposure, and after this time, decreases with increasing time in the salt solution but is still much better than in the coatings with the empty nanocontainers. As the time reaches 60 h, the nanocontainers deplete, leaving behind nanocontainers with fewer inhibitors reducing their performance. This behavior suggests the “self-healing” effect that offers the filled nanocontainers with 5-ATDT [5]. The phase angle vs. ω measurements expresses the barrier phenomenon in these samples (Figure 5). Several organic compounds have demonstrated a significant effect in inhibiting aqueous corrosion in various metals and alloys [32,33]. For example, 5-ATDT forms a membrane on the iron surface, acting as a diffusion barrier excluding the reaction of water to take place [32,34,35]. Inhibitors consisting of nitrogen and sulfur in their structures are more efficient than those having only sulfur or nitrogen [36]. Others developed chelating compounds on zinc surfaces [37]. Inhibitors containing sulfur and nitrogen, such as the 5-ATDT, perform outstandingly on aggressive media [36]. In a different study, the formation of chelate compounds onto the copper substrate was reported when 8-hydroxyquinoline was freed from the TiO_2_ nanocontainers [20]. This study requires combined EIS, Raman spectroscopy, and quantum mechanical calculation to reproduce the spectroscopic data, which proved the creation of the observed chelate structure on the cupper substrate. A similar study in the ORMOSIL coatings with CeO_2_ (5-ATDT) will also need the employment of Raman and EIS spectroscopies to precisely determine the kind of barrier formed on our HDG substrate. This additional work is out of the scope of the present paper. Now, one can base the interpretation of our observations on the great literature work via a possible 5-ATDT protective coating creation. In our work, the 5-ATDT comes from the release from the charged CeO_2_ nanocontainers in the ORMOSIL coatings contributing to the self-healing phenomenon observed in our samples (Figure 6). 

To summarize, the development of ORMOSIL coatings loaded with inhibitors nanocontainers offers significant protection to HDG, making it more usable in the harsh environment described in the introduction. In this work, a complete description of the chemistry of ORMOSIL and nanocontainer production was extensively given to the industry and others in order to reproduce our work. 

## 4. Conclusions

New technology is presented for the protection of HDG against corrosion used in concrete offered by the filled CeO_2_ ceramic nanocontainers with 5-ATDT. The development of the CeO_2_ ceramic nanocontainers follows the established three-step process described in detail in this paper. Then, the nanocontainers were filled with the 5-ATDT corrosion inhibitor at 22%. Next, these nanocontainer systems were incorporated into ORMOSIL coatings. Finally, the anticorrosion properties of all coatings were studied by electrochemical impedance spectroscopy (EIS) using a corrosive environment NaCl of 0.5 M at ambient temperature. As a result, ORMOSIL coating with CeO_2_ (5-ATDT) demonstrated improved properties compared to galvanized steel, ORMOSIL, and ORMOSIL + CeO_2_ (EMPTY) coatings. In addition, the ORMOSIL coating with CeO_2_ (5-ATDT) possesses a self-healing capability.

## Figures and Tables

**Table 1:** The conditions used for the manufacture of polystyrene nanospheres at 80 °C.

Material	Quantity (g)
Styrene	3.62
Potassium persulfate (KPS)	0.3
Sodium dodecyl sulphate (SDS)	0.09
Water	250
SEM (nm)	150 +/− 10

**Table 2:** The conditions used for the manufacture of coated polystyrene nanospheres. The size determined by TEM.

Material	Quantity (g)
Polystyrene (PS)	~0.280
Polyvinylpyrrolidone with average molecular weight 55,000 (PVP)	0.4
Cerium(III) acetylacetonate	0.667
Water	40
Nanosphere size (nm)	180 +/− 10

**Table 3:** Nanosphere sizes after the first (Figure 2a) and second (Figure 2b) heat treatment at 600 °C.

Nanospheres	Size (nm)
Polystyrene	150 +/− 10
Coated Polystyrene	180 +/− 10
(a) Nanocontainers CeO_2_	206 +/− 10
(b) Nanocontainers CeO_2_	140 +/− 10

**Table 4:** The conditions for the composition of the coating.

Material	Quantity (g)
*N*-(2-aminoethyl)-3-(trimethylcyrit)propylamine	3.6
Epoxy resin	41.68
2,2′- diaminediethylamine	4.44
Absolute ethanol	120
Acetone	90

## Data Availability

Not applicable.
